# Bacterial Volatile Organic Compounds as a Strategy to Increase Drought Tolerance in Maize (*Zea mays* L.): Influence on Plant Biochemistry

**DOI:** 10.3390/plants13172456

**Published:** 2024-09-02

**Authors:** Tiago Lopes, Pedro Costa, Paulo Cardoso, Etelvina Figueira

**Affiliations:** 1 Department of Biology, University of Aveiro, Campus Universitário de Santiago, 3810-193 Aveiro, Portugal; tslopes@ua.pt (T.L.); pedrommrscosta@ua.pt (P.C.); pjcardoso@ua.pt (P.C.); 2 CESAM—Centre for Environmental and Marine Studies, University of Aveiro, 3810-193 Aveiro, Portugal

**Keywords:** Bacterial Volatile Organic Compounds (BVOCs), drought, biochemical endpoints, antioxidant response, osmolyte production

## Abstract

Maize is highly susceptible to drought, which affects growth and yield. This study investigated how bacterial volatile organic compounds (BVOCs) affect maize drought tolerance. Drought reduced shoot size but increased root length, an adaptation for accessing deeper soil moisture. BVOCs from strain D12 significantly increased root length and shoot growth under drought conditions. Drought also altered root biochemistry, decreasing enzyme activity, and increased osmolyte levels. BVOCs from strains F11 and FS4-14 further increased osmolyte levels but did not protect membranes from oxidative damage, while BVOCs from strains D12 and D7 strains reduced osmolyte levels and cell damage. In shoots, drought increased the levels of osmolytes and oxidative stress markers. BVOCs from FS4-14 had minimal effects on shoot biochemistry. BVOCs from D12 and F11 partially restored metabolic activity but did not reduce cell damage. BVOCs from D7 reduced metabolic activity and cell damage. These results suggest that BVOCs can modulate the biochemical response of maize to drought, with some strains evidencing the potential to enhance drought tolerance.

## 1. Introduction

The global agricultural landscape is undergoing significant changes driven by the need to meet the food demands of a rapidly growing population while coping with the adverse effects of climate change. Among the various challenges, drought is a preeminent stressor that significantly affects crop productivity, leading to severe economic losses and food insecurity [1,2,3]. Traditional agricultural practices, often based on extensive chemical fertilizers and irrigation use, are increasingly recognized as unsustainable and environmentally damaging [4,5]. Thus, there is an urgent need for innovative and environmentally friendly approaches that can enhance crop resilience and ensure sustainable agricultural productivity.

In this context, bacterial volatile organic compounds (BVOCs) have emerged as promising bioagents with the potential to change modern agricultural practices. BVOCs are a diverse group of low molecular weight compounds emitted by bacterial species that are critical in mediating plant–microbe interactions [6]. These volatiles have been shown to influence plant growth, development, and stress responses, making them attractive candidates for sustainable crop management strategies. Several studies have highlighted the multiple roles of BVOCs in promoting plant health. For example, Ryu et al. [7] demonstrated that specific BVOCs could enhance plant growth by modulating hormonal pathways such as auxin and cytokinin signaling. In addition, Kai et al. [8] reported that BVOCs emitted by rhizobacteria can suppress the growth of soil-borne pathogens, thereby protecting plants from disease. These findings underscore the potential of BVOCs as natural growth promoters and biocontrol agents for agriculture.

One of the most compelling applications of BVOCs is their ability to enhance plant tolerance to abiotic stresses, particularly drought [9,10,11,12]. Drought stress, characterized by limited water availability, triggers a cascade of physiological and biochemical changes in plants, resulting in reduced photosynthetic efficiency, impaired growth, and reduced yield [13,14,15]. Emerging evidence suggests BVOCs may mitigate these adverse effects by inducing plant drought tolerance mechanisms. For example, certain BVOCs have been shown to regulate stomatal closure, thereby reducing water loss through transpiration [16]. Others have been found to improve root architecture, thus enhancing water uptake and nutrient acquisition [17,18].

Maize (*Zea mays*), a staple crop of global importance, is particularly vulnerable to drought stress [19,20]. Therefore, improving the drought tolerance of maize is critical for maintaining yields and food security, especially in regions with a maize-based diet and those prone to water scarcity. This study explores maize plant’s response (growth and biochemistry) to BVOC blends produced by different bacteria. By studying biometric parameters (length and weight), cellular damage (lipid peroxidation (LPO) and protein carbonylation (PC)), metabolic capacity (protein content and electron transport system (ETS)), osmotolerance (sugars and proline), and antioxidant (superoxide dismutase (SOD) and catalase (CAT)), and biotransformation (glutathione S-transferases (GSTs)) response in both shoots and roots, we aim to provide a comprehensive assessment of the efficiency of BVOCs in promoting drought tolerance in maize.

## 2. Results

### 2.1. Growth and Biochemical Alterations in Roots

**Figure 1:**
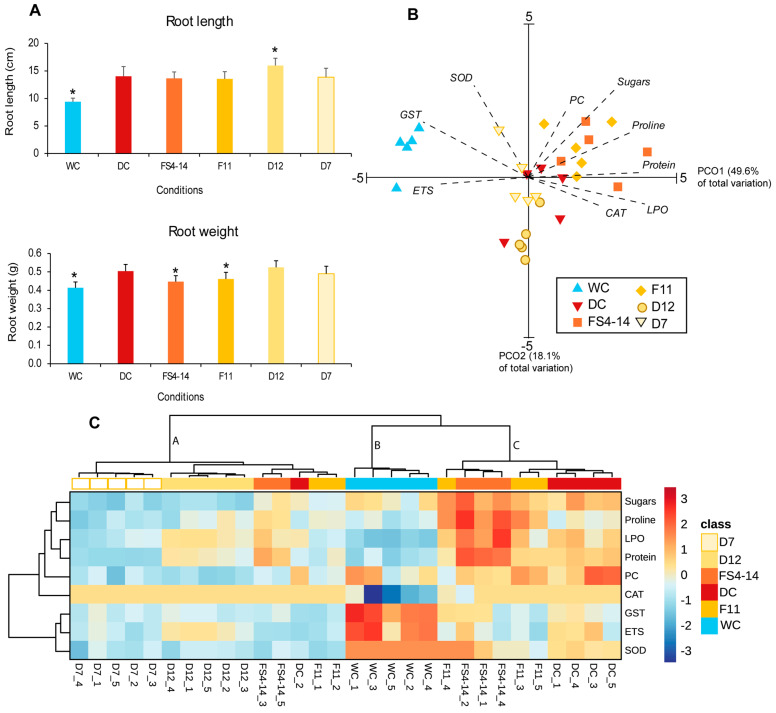
Effect of bacterial volatiles on the roots of drought-stressed maize plants. Plants were simultaneously exposed to drought and bacterial volatile compounds (BVOCs) from four bacteria (D7, D12, FS4-14, F11); drought (DC) and watered (WC) plants not exposed to BVOCs were also included. (**A**) Root length and weight. Values are means of eight replicates ± standard deviation. Asterisks (*) indicate significant differences (*p* < 0.05) with DC. (**B**) Principal coordinate ordination (PCO) of biochemical parameters for the conditions tested; parameters with a higher contribution (r ≥ 0.65) were superimposed on the graph (Prot—protein, ETS—electron transport system activity, SOD—superoxide dismutase activity, CAT—catalase activity, GSTs—glutathione S-transferases activity, LPO—lipid peroxidation, PC—protein carbonylation, Sugars—sugars, Proline—proline). (**C**) Heatmap of the biochemical parameters for each condition. The scale represents z-scores. Here, antioxidant activity, protein, and chlorophyll content are represented by orange to red colors; lower antioxidant activity, protein, and chlorophyll content are represented by light blue to dark blue. Five replicates per condition were used for B and C.

Drought (DC) increased the length and weight of plant roots compared with plants not exposed to drought (WC). Exposure to the BVOCs from three bacterial strains (FS4-14, F11 and D7) did not affect the length of roots in drought conditions (Figure 1A), but BVOCs from D12 significantly increased root length compared to DC. The effect of BVOCs on root weight varied among strains, with two (FS4-14 and F11) significantly reducing weight, one (D12) increasing (4.1%) weight non-significantly, and D7 having no effect compared to DC.

Principal component ordination (PCO) analysis (Figure 1B) of the biochemical determinants tested for each condition showed that PCO1 and PCO2 together explained 67.7% of the total variation. Along PCO1 (49.6%), two groups were formed. The watered condition (WC) was located on the negative side, and drought conditions were mostly on the positive side of the axis. In addition, DC, D7, and D12 were located near the axis origin, and FS4-14 and F11 were located on the most positive side of axis 1. PCO2 (18.1%) separated the conditions exposed to drought, with DC and D7 near the origin of the axis, D12 on the negative side, and FS4-14 and F11 on the positive side. From the PCO analysis, ETS, GSTs, and SOD were highly correlated with WC; proline, sugars, LPO, proteins, and PC were more correlated with FS4-14 and F11; and CAT was correlated with D12 (Supplementary Table S2). 

The heatmap (Figure 1C) evidences drought (DC) as a driver of major changes in root biochemistry, with increases in osmolytes (proline and sugars), antioxidant (CAT) activity, and damage (LPO and PC), but reductions in metabolic capacity (ETS), SOD activity, and biotransformation (GSTs) capacity compared to the roots of watered plants (WC). The volatiles of FS4-14 and F11 had little impact on root biochemistry compared with DC; therefore, these three conditions clustered together (cluster C). However, these two strains increased the levels of osmolytes, mainly proline, and the level of proteins (especially FS4-14); reduced protein damage (PC); and increased membrane damage (LPO) compared to DC. BVOCs of D7 and D12 had a distinct effect on root biochemistry, as these plants form a cluster (cluster A) separated from the remaining conditions, including drought and watered plants (clusters B and C). Except for CAT activity, which is higher than WC, the remaining parameters, especially in D7, show osmolyte and damage values similar to WC (cluster B) and lower than those in DC, FS4-14, and F11 (cluster C). Additionally, they exhibit lower metabolic and antioxidant activity (ETS, SOD, and GSTs) than the conditions in cluster B and especially cluster C.

### 2.2. Growth and Biochemical Alterations in Shoots

**Figure 2:**
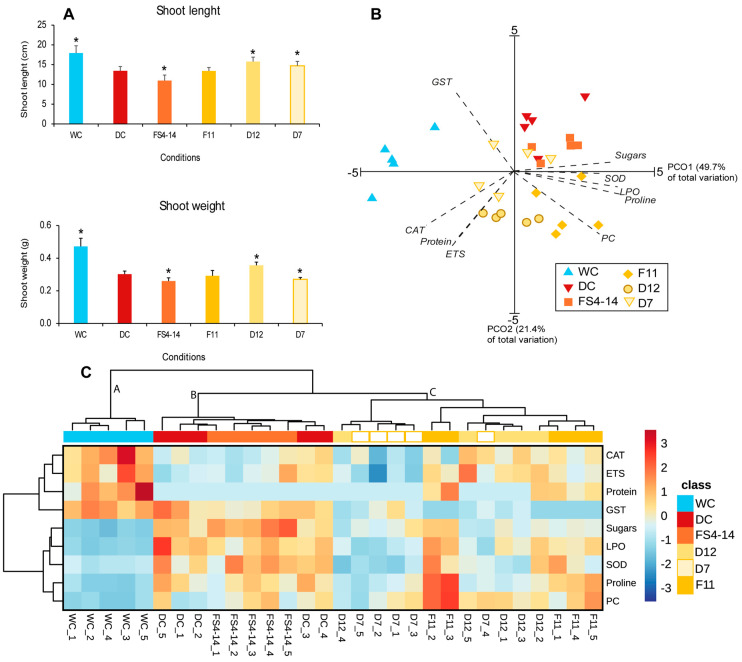
Effect of bacterial volatiles on shoots of drought-stressed maize plants. Plants were simultaneously exposed to drought and bacterial volatile compounds (BVOCs) from four bacteria (D7, D12, FS4-14, F11); drought (DC) and watered (WC) plants not exposed to BVOCs were also included. (**A**) Shoot length and weight. Values are means of eight replicates ± standard deviation. Asterisks (*) indicate significant differences (*p* < 0.05) with DC. (**B**) Principal coordinate ordination (PCO) of biochemical parameters for the conditions tested; parameters with a higher contribution (r ≥ 0.65) were superimposed on the graph (Prot—protein, ETS—electron transport system activity, SOD—superoxide dismutase activity, CAT—catalase activity, GSTs—glutathione S-transferases activity, LPO—lipid peroxidation, PC—protein carbonylation, Sugars—sugars, Proline—proline). (**C**) Heatmap of the biochemical parameters for each condition. The scale represents z-scores. Higher antioxidant activity, protein, and chlorophyll content are represented by orange to red colors; lower antioxidant activity, protein, and chlorophyll content are represented by light blue to dark blue. Five replicates per condition were used for B and C.

In contrast to the roots, drought (DC) significantly decreased shoot weight and length (Figure 2A). Exposure to BVOCs impacted shoot growth differently, with FS4-14 significantly aggravating the drought effect, and D12 and D7 significantly alleviating the drought effect. F11 had an insignificant effect.

Principal component ordination (PCO) analysis (Figure 2B) of the biochemical determinants tested for each condition showed that PCO1 and PCO2 together explained 71.1% of the total variation. Along PCO1 (49.7%), two groups were formed, with the watered condition (WC condition) on the far negative side and the drought conditions mostly on the positive side of axis 1. PCO2 (21.4%) separated the effects of BVOCs and drought on shoot biochemistry, with DC and FS4-14 on the positive side, D7 near the axis 2 origin, and D12 and F11 on the negative side of the axis. PCO analysis revealed that GSTs, CAT, ETS, and proteins were more strongly correlated with WC; sugars, SOD, and LPO correlated with DC and FS4-14; and PC correlated with F11 and D12 (Supplementary Table S3). 

The heatmap analysis (Figure 2C) showed that drought was the main driver of change in the shoot, with clusters B and C containing drought conditions exposed or not to BVOCs and watered plants (WC) forming a different cluster (cluster A). Cluster A (WC) is characterized by high metabolic (ETS) activity, high GSTs and CAT activity, high protein content, low levels of osmolytes, low cell damage, and low SOD activity. Drought caused the accumulation of osmolytes (proline and sugars), SOD activity, and membrane damage (LPO). Exposure to FS4-14 BVOCs had little influence on shoot biochemistry compared with DC, and these two conditions clustered together (cluster B). The remaining conditions (D12, D7, and F11) clustered (cluster C) with osmolyte concentration and enzyme activity values intermediate between clusters A and B. This cluster includes condition D7, which presents lower metabolic and enzymatic activities and lower concentrations of metabolites than the remaining conditions tested.

## 3. Discussion

Maize is very susceptible to drought damage due to the inability to delay vegetative growth, and higher yields will only be obtained if environmental conditions are favorable at all stages of growth [19,21]. This study tackled maize tolerance to drought by investigating plants’ responses to BVOCs exposure. The drought harmed plants, significantly reducing shoot size, but BVOCs changed the plant response to drought anatomically and biochemically. Plant anatomy was changed, with shorter shoots and longer roots observed. This adaptation is described in several studies as an effective way for plants to access deeper layers of moister soil [22,23,24,25,26]. 

Inoculation with PGPB has been reported to promote root elongation and increase drought tolerance in plants but is generally associated with the ability of bacteria to produce phytohormones, especially auxins. However, BVOCs, such as 2-phenylethanol, 1-hexanol, acetoin, 2-hexenal, 1-octen-3-ol, limonene, and 2,3-butanediol, have been described to promote root elongation through different mechanisms, including modulating plant hormone levels and activating stress response pathways, thereby indirectly promoting root growth and improving plant’s ability to uptake water and nutrients from the soil [23,24,27,28,29,30].

However, these studies were not carried out under drought conditions. Our study showed that BVOCs produced by the D12 strain promoted root length under drought conditions, which may allow for more effective water absorption and lower water stress levels. Indeed, our results show higher growth of shoots in condition D12 than in other drought conditions.

Drought also changed the biochemistry of plants both below and aboveground. In roots, drought decreased enzyme and metabolic activities and increased osmolyte concentrations. A higher concentration of osmolytes reduces the water potential of tissues and allows for more efficient water absorption under drought conditions with little impact on cell metabolism [31,32]. Exposure to BVOCs emitted by F11 and FS4-14 had little impact on the enzymatic and metabolic activities of roots but increased osmolytes, especially proline, thus protecting proteins from oxidative damage but failing to protect membranes from oxidative damage. Proline maintains the hydration of globular proteins, preventing their precipitation and preserving their conformation and activity [33,34]. This action was pointed out by Cruz et al. [34] as the main mechanism of tolerance of maize plants to salinity. The metabolic effort to produce osmolytes reduces the resources available for growth, and the roots and shoots of conditions F11 and FS4-14 were significantly smaller than those of DC.

The biochemistry of roots under the influence of BVOCs produced by D12 and D7 differs greatly from other conditions. The BVOCs of these two strains induced longer roots, increasing water absorption, reducing water stress, and the need for osmoregulation. In fact, these plants had lower osmolyte levels than other drought conditions and similar levels to WC. Furthermore, they reduced metabolic activity, with the electron transport chain being one of the main sites for generating reactive oxygen species (ROS) [33,35]. A reduction in ROS production will consequently mitigate cellular damage.. Our results corroborate this assumption since cell damage decreased, especially in proteins. Lower exposure of proteins to ROS decreases their degradation and reduces the need to replace degraded, less-functional proteins [36,37]. This conservative metabolism reduced the metabolic rate and spent fewer resources on metabolic and osmotic adaptation, leaving resources for growth.

As noted in the roots, the shoots of watered plants showed high metabolic activity, high levels of proteins and GSTs activity, less cell damage, and lower osmolyte concentrations. Drought reverted this pattern, with only GSTs activity remaining high. Lopes et al. [38], when exposing maize plants to drought, found that most of the biochemical parameters analyzed did not change between drought and watered plants (sugars, ETS, SOD, and LPO) or the increase caused by drought (proline, proteins) was not significant. The divergence from our results is not due to the stress imposed but perhaps to the different growth conditions [39,40,41,42]. BVOCs produced by FS4-14 did not induce major biochemical differences compared to DC, except for an evident increase in sugar levels. The effort required for this adaptation seems enough for plants to grow significantly less than that required for DC.

BVOCs of D12 and F11 restored, at least in part, the lower metabolic activity, protein levels, and CAT activity induced by drought. However, they had little effect on cellular damage, which may be related to their higher metabolic rate, ROS production, and lower GSTs activity. ROS react with unsaturated fatty acids and produce lipid peroxides [43]. Much of the oxidative damage is caused by lipid peroxidation products, such as aldehydes, which damage important cell molecules, such as proteins [44]. GSTs convert aldehydes into less toxic compounds, protecting proteins from oxidation [45]. The lower GSTs activity in the D12 and F11 conditions could not protect proteins from damage, and high protein carbonylation levels were observed. D7 highlights a strategy similar to that found in the root: reduced metabolic activity, lower ROS production, and less cell damage. This strategy was inefficient since the shoot weight was significantly lower than that of DC.

## 4. Material and Methods

### 4.1. Bacterial Strains

Bacteria were isolated from the roots of the endemic plants (*Tetraena simplex*, *Tetraena stapffi* and *Stipagrostis* sp.) from the Namib desert (15°08′06.2″ S 12°12′51.7″ E) and (*Zea mays*) Coruche, Portugal (38°56′41.637″ N–8°30′44.554″ W). Five 1 cm root sections from each root system were surface sterilized by soaking in 96% ethanol for 5 s and then immersed in a 30% hydrogen peroxide solution for 2 min. Root sections were thoroughly rinsed twice with sterile deionized water and crushed. The macerated material was streaked onto yeast extract-mannitol (YMA) plates. After 10 days, morphologically distinct single colonies were re-streaked and preserved in 15% glycerol at −80 °C. Identification at the genus level was performed using partial 16S rRNA gene sequencing, and the sequences were submitted to GenBank (PQ201073, OR948275.1) [46]. Four bacterial strains (FS4-14, F11, D12, and D7) were selected based on their osmotolerance and plant growth-promoting abilities (Table S1). 

### 4.2. Plant Experiment

To assess the effects of volatiles released by different bacteria on drought-stressed *Zea mays* plants, seeds (Aquamax P9911) were sterilized (70% ethanol, 5% bleach, and washed in sterilized distilled water) and germinated for two days under sterile, moist, and dark conditions. A two-day-old maize seedling was planted in sterile sealed jars (16.5 × 7.5 cm Ø) containing 200 g of previously autoclaved dried sand. The sand’s water-holding capacity (WHC) inside the jars was 5% (50% growth inhibition). A 3 cm Ø sterile dish containing 4.5 mL yeast mannitol agar medium (no induced drought-stress) was included on the surface of the sand in the jar and immediately inoculated with 100 µL of 10^8^ cells/mL fresh culture of each bacterial strain. Jars were closed with lids and sealed with parafilm to avoid BVOC loss. A dry (5% WHC) non-inoculated control (DC) and a wet non-inoculated control (35% WHC) were included. Each condition (four bacterial strains and the two controls) was 8-fold replicated. Jars were maintained for 7 days in a greenhouse chamber with a 16 h photoperiod of light (1450 μmol/m²/s) at 26 ± 1 °C and 18 ± 1 °C during dark. At the end of the experiment, plants were collected, and the roots were separated from the shoots. The roots were washed with deionized water to remove the attached sand, and excess water was removed with absorbent paper. The roots and shoots were measured, weighed, and stored at −80 °C for further biochemical analysis.

### 4.3. Biochemical Analysis 

#### 4.3.1. Extraction

For the assessment of the parameters of ETS, protein content, SOD, CAT, GSTs, LPO, PC, and sugars, frozen samples (roots and shoots) were first homogenized by mortar and pestle in liquid nitrogen and then using an ultrasonic probe (for 30 s at 0.6 Hz) in sodium phosphate buffer (1:2 (*w*/*v*) (50 mM sodium dihydrogen phosphate monohydrate (KH_2_PO_4_), 50 mM disodium hydrogen phosphate dihydrate (K_2_HPO_4_), 1 mM ethylenediaminetetraacetic acid disodium salt dihydrate (EDTA), 1% (*v*/*v*) Triton X-100, and 1 mM dithiothreitol (DTT), pH 7.0) [38]. Homogenates were centrifuged at 3000× *g* for 3 min at 4 °C and immediately used for electron transport system (ETS) determination. For other parameters (protein content, SOD activity, CAT activity, PC, GSTs activity, and sugar content), homogenates were centrifuged at 12,000× *g* for 10 min at 4 °C and stored at −80 °C until use. LPO was measured in the homogenate pellet.

For proline, frozen samples (roots and shoots) were first homogenized in liquid nitrogen using a mortar and pestle, followed by homogenization in 3% sulfosalicylic acid using an ultrasonic probe (for 30 s at 0.6 Hz) and centrifugation at 12,000× *g* for 10 min at 4 °C.

#### 4.3.2. Biochemical Assays

ETS activity was determined according to the method proposed by King and Packard [47], with modifications by De Coens and Janssen [48]. Samples were mixed with balanced salt solution (BSS) buffer (0.13 M Tris-HCl, 0.3% (*v*/*v*) Triton X-100, pH 8.5), 1.7 mM NADH, 250 µM NADPH, and 8 mM p-iodonitrotetrazolium (INT). Absorbance was measured at 490 nm for 10 min, with readings taken at intervals of 25 s. The molar extinction coefficient of formazan (ε = 15,900 M^−1^ cm^−1^) was used, and results were expressed as nanomoles of formazan per minute per gram of fresh weight (nmol/min/g FW).

Protein content was measured using the Biuret reaction method described by Robinson and Hodgen [49]. Samples were mixed with the biuret reaction solution in a volume ratio 1:6 and incubated for 10 min at room temperature in the dark. Absorbance was recorded at 540 nm using bovine serum albumin (BSA) standards (0–1 mg/mL). Results were expressed in milligrams of protein per gram of fresh weight (mg/g FW).

SOD activity was measured according to the method described by Beauchamp and Fridovich [50]. Samples were mixed with the reaction buffer (50 mM Tris-HCl (pH 8), 0.1 mM diethylenetriaminepentaacetic acid (DTPA), 0.1 mM hypoxanthine, and 68.4 µM nitroblue tetrazolium) and 51.6 mU/mL xanthine oxidase in a ratio of 1:10:1 (*v*/*v*/*v*). This mixture was incubated for 20 min at room temperature on an orbital shaker, and the absorbance was recorded at 560 nm. Results were expressed as enzyme units per gram of fresh weight (U/g FW).

CAT activity was determined according to the method described by Johansson and Borg [51]. Samples were mixed with the reaction buffer (50 mM K₂HPO₄ and KH₂PO₄ (pH 7.0), methanol (reagent grade), and 35.28 mM hydrogen peroxide). The mixture was then incubated at room temperature for 20 min. After this incubation, 10 M potassium hydroxide (KOH) and 34.2 mM Purpald were added, followed by another 10 min incubation. Finally, 65.2 mM potassium periodate was added, and the mixture was incubated for another 10 min. The absorbance was measured at 540 nm. Formaldehyde standards (0–20 µM) were used, and the results were expressed as enzyme units per gram of fresh weight (U/g FW).

GSTs activity was evaluated according to the method described by Habig et al. [52]. Samples were mixed with the reaction solution (0.1 M K₂HPO₄ and 0.1 M KH₂PO₄ (pH 6.5), 60 mM 1-chloro-2,4-dinitrobenzene (CDNB), and 10 mM glutathione (GSH) (ratio 1:2)), and absorbance was measured at 340 nm for 10 min with readings taken at intervals of 25 s. The molar coefficient extinction of thioether (ε = 9.6 mM^−1^ cm^−1^) was used, and results were expressed in milliunits of enzyme per gram of fresh weight (mU/g FW).

LPO was evaluated using the method described by Buege and Aust [53]. Samples were mixed with 0.5% 2-thiobarbituric acid and 20% trichloroacetic acid in a ratio of 1:3:3 (*w*:*v*:*v*) and incubated at 96 °C for 25 min. The reaction was stopped by placing the samples on ice. Centrifugation was performed at 5000× *g* for 5 min at 4 °C. The absorbance was measured at 532 nm, and the molar extinction coefficient of malondialdehyde (MDA) (ε = 1.56 × 10^5^ M^−1^ cm^−1^) was used for calculations. Results were expressed as nanomoles of MDA equivalents per gram of fresh weight (nmol/g FW).

PC was quantified using the method described by Mesquita et al. [54]. Samples were mixed with 10 mM 2,4-dinitrophenylhydrazine (DNPH) and incubated for 10 min. After, 6 M sodium hydroxide (NaOH) was added at a ratio of 2:2:1 (*v*/*v*/*v*), and the mixture was incubated for another 10 min. Absorbance was measured at 450 nm using the DNPH extinction coefficient (ε = 22,308 M^−1^ cm^−1^), and the results were expressed as micromoles per gram of fresh weight (µmol/g FW).

Sugar content was quantified according to the method described by Dubois et al. [55]. Samples were mixed with 5% phenol and 98% sulfuric acid and incubated for 45 min at room temperature. After centrifugation at 5000× *g* for 5 min at 4 °C, the absorbance was measured at 492 nm. Glucose standards ranging from 0 to 1 mg/mL were used, and the results were expressed as milligrams per gram of fresh weight (mg/g FW).

Proline content was quantified according to the method described by Bates et al. [56], in which the supernatant was mixed with acid ninhydrin and glacial acetic acid (reagent grade) in a ratio of 1:1:1 (*v*/*v*/*v*) and incubated at 100 °C for 45 min. The reaction was stopped by placing the samples on ice, and the absorbance was measured at 520 nm. Proline standards (0–100 μg/mL) were used, and the results were expressed as micrograms of proline per gram of fresh weight (μg/g FW). For each condition, five replicates were used.

### 4.4. Statistical Analysis

Hypothesis testing for biometrics and biochemical parameters was performed using a one-factor nonparametric permutational analysis of variance. The analysis was performed using Primer v.6 with the Permanova add-on [57].

The dataset was subject to a square root transformation, a similarity matrix using an Euclidean distance metric, and a Monte Carlo test (9999 permutations). Pairwise comparisons assessed significance, and differences were considered significant only at *p* < 0.05 and marked with an asterisk (*) in the figures and tables. The null hypothesis tested was that there would be no differences between the non-inoculated drought-stressed plants and the other conditions (watered plants and inoculated drought-stressed plants).

To understand the biochemical changes induced by inoculated bacteria in drought-stressed plants, principal component ordination (PCO) was performed using the data obtained from the biochemical assays. The dataset followed a square-root transformation and was used to construct an Euclidean matrix. Pearson correlation vectors (correlation ≥ 0.65) were inserted into the PCO graph to better identify the parameters that had the most influence on plant biochemistry among the different tested conditions.

Heatmaps illustrating the biochemical parameters for each condition were generated using MetaboAnalyst 5.0 [58]. The data were subjected to autoscaling and standardization using the median, and the z-scores were subsequently calculated. For each cell, the z-score was calculated by subtracting the mean and dividing it by the standard deviation. A higher z-score reflects higher antioxidant activity and protein and chlorophyll content. Additionally, the data were employed to construct an Euclidean matrix, and clusters were constructed using Ward’s linkage (the minimum variance method).

## 5. Conclusions

Our study showed the influence of BVOCs on the anatomy and biochemistry of plants under drought conditions. This influence varied among bacterial strains and depended on the blend of BVOCs produced by each strain. Knowing the specific effects of the BVOCs of each strain may enable the creation of specific blends to maximize the effects on plant tolerance to drought and putatively to other stresses, leading to new methodologies for more efficient water use and, thus, more sustainable production systems. However, there is work to be done to identify the compounds, determine the most effective dosages, and determine the most appropriate form and time for their application before their use can effectively mitigate the effect of drought in crop fields.

## Data Availability

Dataset available on request from the authors.

## Supplementary Materials

The following supporting information can be downloaded at: https://www.mdpi.com/article/10.3390/plants13172456/s1, Table S1. Identification, osmotolerance and Plant Growth Promotion (PGP) traits (siderophore production, phosphate and potassium solubilization, alginate, proline, and indole-3-acetic acid synthesis) of selected bacteria isolated from the roots of plants grown in arid environments; Table S2 Roots: Effect of volatiles produced by bacterial strains isolated from a desert environment on the root growth (fresh weight and length) and biochemistry (protein, electron transport system—ETS, superoxide dismutase—SOD, catalase—CAT, glutathione S-transferases—GSTs, protein carbonylation—PC, lipid peroxidation—LPO, proline, sugars) of drought stressed maize plants; Table S3 Shoots: Effect of volatiles produced by bacterial strains isolated from a desert environment on the shoot growth (fresh weight and length) and biochemistry (protein, electron transport system—ETS, superoxide dismutase—SOD, catalase—CAT, glutathione S-transferases—GSTs, protein carbonylation—PC, lipid peroxidation—LPO, proline, sugars) of drought stressed maize plants.
