# Supplementary material for: Bacterial Volatile Organic Compounds as a Strategy to Increase Drought Tolerance in Maize (Zea mays L.): Influence on Plant Biochemistry
Source: Plants (Basel). 2024 Sep 2;13(17):2456. doi: 10.3390/plants13172456 (PMC11397109; doi:10.3390/plants13172456)
Supplement: Supplementary file 1 [file plants-13-02456-s001.zip › plants-3161448-supplementary.pdf]

Table S1. Identification, osmotolerance and Plant Growth Promotion (PGP) traits (siderophore production, phosphate and potassium solubilization, alginate, proline, and indole-3-acetic acid synthesis) of selected bacteria isolated from the roots of plants grown in arid environments.

Strain	Identification	Assession number	Osmotolerance IC50 (%)	Siderophores (Ø halo/colony)	Phosphate solubilization (Ø halo/colony)	Potassium solubilization (Ø halo/colony)	Alginate (PEG 10%) µg /OD	IAA (PEG 10%) µg /OD	Proline (PEG 10%) µg /OD
D12	Unknown	-	15	1.07	1.54	-	38.56	197.02	10.69
D7	Unknown	-	15	1.33	1.71	4.86	35.53	167.49	46.87
F11	*Siccibacter*	PQ201073	12	1.5	1.63	-	37.61	139.87	12.48
FS4-14	*Acinetobacter*	OR948275.1	12	2.16	0.50	-	-	-	18.10

Table S2 Roots: Effect of volatiles produced bybacterial strains isolated from a desert environment on the root growth (fresh weight and length) and biochemistry (protein, electron transport system - ETS, superoxide dismutase - SOD, catalase - CAT, glutathione S-transferases - GSTs, protein carbonylation - PC, lipid peroxidation - LPO, proline, sugars) of drought stressed maize plants. Dry (DC) and watered (WC) non-inoculated conditions were included in the experiment. Values are means of 5 replicates ± standard deviation. Significant differences ($p < 0.05$) relatively to DC are marked with asteriscs (*).

Parameters	Conditions				
	WC	DC	FS4-14	F11	D12	D7

Parameters	WC	DC	FS4-14	F11	D12	D7
Protein (mg/ g FW)	3.31 ± 0.53 *	5.65 ± 0.74	8.47 ± 1.35 *	6.55 ± 1.08	6.00 ± 1.01	5.05 ± 0.58 *
ETS (nmol/min/g FW)	537.3 ± 69.2 *	406.1 ± 63.8	356.5 ± 75.6	377.3 ± 55.7	445.5 ± 91.3 *	467.5 ± 72.8 *
SOD (U/ g FW)	10.82 ± 0.90 *	8.32 ± 0.63	9.43 ± 1.06 *	9.58 ± 1.28	8.41 ± 0.77	10.13 ± 1.52
CAT (U/ g FW)	6.99 ± 1.93 *	13.08 ± 3.10	12.67 ± 2.89	14.16 ± 2.81 *	13.24 ± 2.19 *	16.63 ± 2.19 *
GSTs (mU/ g FW)	79.82 ± 8.00 *	33.75 ± 9.21	40.25 ± 10.70	35.95 ± 10.20	41.64 ± 9.41	43.24 ± 8.25
PC (µmol/ g FW)	1.58 ± 0.29	1.93 ± 0.41	1.54 ± 0.33	1.95 ± 0.43	1.37 ± 0.37 *	1.81 ± 0.40
LPO (nmol/ g FW)	0.47 ± 0.05 *	1.16 ± 0.13	1.58 ± 0.38 *	1.18 ± 0.20	1.34 ± 0.22	1.13 ± 0.15
Proline (µg/ g FW)	23.83 ± 2.51	26.77 ± 3.51	46.84 ± 6.85 *	46.49 ± 2.64 *	33.25 ± 5.52 *	31.73 ± 5.00
Sugars (mg/ g FW)	40.34 ± 5.25	45.41 ± 5.50	55.54 ± 4.12 *	53.10 ± 5.54 *	40.04 ± 4.85 *	43.91 ± 3.22

Table S3 Shoots: Effect of volatiles produced bybacterial strains isolated from a desert environment on the shoot growth (fresh weight and length) and biochemistry (protein, electron transport system - ETS, superoxide dismutase - SOD, catalase - CAT, glutathione S-transferases - GSTs, protein carbonylation - PC, lipid peroxidation - LPO, proline, sugars) of drought stressed maize plants. Dry (DC) and watered (WC) non-inoculated conditions were included in the experiment. Values are means of 5 replicates ± standard deviation. Significant differences ($p < 0.05$) relatively to DC are marked with asteriscs (*).

Parameters	Conditions											
	WC		DC		FS4-14		F11		D12		D7	
Protein (mg/ g FW)	34.22 ± 5.03	*	22.93 ± 1.51		25.60 ± 2.24		31.23 ± 4.91	*	31.15 ± 3.13	*	30.93 ± 3.57	*
ETS (nmol/min/g FW)	478.9 ± 70.2	*	346.4 ± 64.2		398.8 ± 56.8		408.9 ± 35.5		500.4 ± 79.9	*	373.8 ± 60.2	
SOD (U/ g FW)	8.28 ± 0.81	*	10.59 ± 1.02		11.91 ± 1.72		11.17 ± 1.19		11.46 ± 3.02		10.88 ± 2.75	
CAT (U/ g FW)	26.92 ± 6.23	*	18.13 ± 3.06		18.32 ± 2.04		19.72 ± 4.07		22.60 ± 4.93	*	20.80 ± 5.41	
GSTs (mU/ g FW)	92.5 ± 15.8		78.6 ± 17.4		62.4 ± 15.5		25.5 ± 3.8	*	39.7 ± 10.1	*	61.8 ± 14.9	
PC (μmol/ g FW)	3.47 ± 0.18	*	5.15 ± 1.13		6.12 ± 1.05		8.51 ± 2.12	*	7.27 ± 0.96	*	7.19 ± 1.34	*
LPO (nmol/ g FW)	3.13 ± 0.37	*	6.50 ± 1.13		6.21 ± 0.82		6.39 ± 0.63		6.33 ± 0.80		4.57 ± 0.46	*
Proline (μg/ g FW)	27.85 ± 4.20	*	61.82 ± 9.81		59.55 ± 7.34		86.71 ± 16.16	*	50.78 ± 6.76		53.53 ± 8.29	
Sugars (mg/ g FW)	22.59 ± 2.09	*	50.47 ± 7.55		65.19 ± 13.38	*	49.31 ± 7.11		45.62 ± 7.78		51.17 ± 8.34	
